# Correlation of Self-Reported Questionnaire (KOOS) with Some Objective Measures in Primary OA Knee Patients

**DOI:** 10.1155/2014/301485

**Published:** 2014-01-16

**Authors:** Kulandaivelan Sivachidambaram, Mahamed Ateef, Shaziya Tahseen

**Affiliations:** ^1^Department of Physiotherapy, GJUST, Hisar, Haryana 125 001, India; ^2^Sainath University, Ranchi, India; ^3^V-Care Physiotherapy Center, Hyderabad 500 035, India

## Abstract

*Purpose*. Objective of the present study was to see the correlation of subjectively measured KOOS questionnaire with objectively measured 6-minute walk test (6-MWT), age, height, weight, and BMI. *Participants*. 251 subjects with OA knee based on American College of Rheumatology criteria. *Methods*. After passing inclusion and exclusion criteria, the following parameters were recorded: age, height, weight, and BMI. Then subjects were asked to fill KOOS questionnaire; then all subjects were asked to do self-paced walk for 6 minutes. *Analysis*. Spearman rank test was done to see the correlation. Significant level was set at *P* < 0.05. *Results*. 6-MWT had a weak correlation with KOOS-ADL (rho 0.461) and strong correlation with KOOS-symptom, KOOS-pain, and KOOS-sports and very strong correlation with KOOS-QOL. BMI had a strong correlation with KOOS-pain, KOOS-symptom, KOOS-ADL, and KOOS-sports and very strong correlation with KOOS-QOL. Weight had a weak correlation with KOOS-symptom, KOOS-ADL, and KOOS-sports and strong correlation with KOOS-pain and KOOS-QOL. All the above values were significant with *P* < 0.001. *Conclusion*. KOOS is strongly positively correlated with 6-MWT and negatively correlated with BMI. Its correlation strength has decreased with weight.

## 1. Introduction

Osteoarthritis (OA) in knee joint is the most common reason behind functional disability that leads to lowered quality of life in old age people of India. Some of the objective factors that may influence OA disease severity include but are not limited to age, height, weight, and BMI. In physiotherapy practice, lowering the pain, other symptoms, improving the activities of daily living (ADL), and quality of life (QOL) are main outcome criteria for OA knee rehabilitation. To achieve this rehabilitative goal, physiotherapists use both self-reported questionnaires as well as performance-based tests to monitor prognosis in the above said parameters, that is, pain, symptoms, ADL, and QOL.

Self-reported questionnaires give subjective information about the disease process without examiner bias within short period of time [1], whereas, performance-based tests objectively measures patient's ability of perform ADL activities. Examples for self-reported questionnaire are WOMAC, SF-36, KOOS, and so forth. Examples for physical performance tests are timed up and go test (TUG), six-minute walk test (6-MWT), stair climbing test, and so forth [2]. Physical performance tests are affected by motivation and not affected by psychogenic factors such as beliefs, expectations, cognitive impairments, and cultural, lingual, and educational level [3, 4].

Possible difference between the two may be performance-based tests measure functional limitation, whereas self-reported questionnaire reveals disability, that is, combination of social and psychological side of the functional limitation. Functional limitation is sooner detected by performance-based tests than self-reported questionnaires [5, 6]. There is no consensus regarding the use of tests while some prefer to use self-reported questionnaires because they are easy to administer and have high internal consistency features; others prefer performance-based tests as they consider that these tests are essential for ADL evaluation of the patient. Ideally, both self-reported and functional tests should be used in knee rehabilitation setting [7, 8].

In order to ascertain more valid results, performance-based tests should always be correlated with self-reported measures. In previous studies, WOMAC, SF-36, and KOOS are compared with timed up and go (TUG) test [7, 9–11]. However there is no study that compares self-reported questionnaire with 6-MWT which is more deals with ADL as this measures both walking ability as well as endurance. KOOS is a relatively newer self-reported questionnaire that was developed from WOMAC by Roos et al. [12] for OA knee patients. Thus, primary objective of present study was to see the correlation of subjectively measured KOOS questionnaire with objectively measured 6-minute walk test (6-MWT), age, height, weight, and BMI.

## 2. Methodology

Present study was a cross-sectional survey study in which data was extracted from individuals with clinical and nonradiographic idiopathic or primary knee OA based on American College of Rheumatology (ACR) criteria [13]. ACR criteria for clinical diagnosis of idiopathic knee OA were based on knee pain in either knee on most days for at least 1 month in the previous year and at least two of the following signs/symptoms: stiffness, crepitus, bony tenderness, and bony enlargement. These items were assessed through clinical examination conducted by a trained clinical physiotherapist. Exclusion criteria were (1) self-reports of currently doing lower-extremity exercise ≥2 times per week; (2) self-reports of currently fitness walking ≥90 minutes per week; (3) being unable to read English/Urdu questionnaire (KOOS); (4) inflammatory arthritis; (5) self-reports of having current knee conditions such as meniscus tears and knee ligament ruptures; (6) fracture of spine or lower extremities; (7) being scheduled to undergo a major surgical procedure in the next 6 months; (8) those with neurological conditions; (9) having contraindications for exercise testing based on American College of Sports Medicine [14] criteria. Total of 251 patients (117 males, 134 females) met the above said criteria and were included for the present study. Their anthropometric characteristics along with ranges are given in Table 1.

### 2.1. Measurements

#### 2.1.1. Knee Injury and Osteoarthritis Outcome Score (KOOS)

KOOS is a self-reported questionnaire consisted of 42 questions in total addressing five patient-related domains including pain (9 questions), other disease-specific symptoms (7 questions), activity of daily living (ADL) (17 questions), sport and recreation function (5 questions), and knee-related quality of life (4 questions). All items were evaluated by five-point Likert scale. Total score changes between 0 and 100. Higher scores indicate better function. It takes 5–10 min to fill in all questions.

#### 2.1.2. 6-Minute Walk Test (6-MWT)

6-MWT was used to study physical performance. The walking track was a 30 m long empty corridor with marks on every 5 meters. Patients were asked to walk back and forth along the 30 m track and to cover the longest possible distance in 6 minutes. At the end of every minute patients were informed without any verbal encouragement. Two weekly test-retest reliability of walking distance was reported to be 0.87 for patients with knee OA [2].

#### 2.1.3. Height (cm)

The Physiotherapist measured height with a stadiometer. Patients stood on a firm surface in their bare feet to have their height measured.

#### 2.1.4. Body Mass (kg) and Body Mass Index

Body mass was measured at the end of examination with a calibrated bathroom-type digital scale, on a firm surface. Patients were weighed in the standing position, wearing light indoor clothes, but no shoes, jewellery, or heavy clothing. Body mass index (BMI) was calculated with the formula (weight/(height)^2^).

### 2.2. Statistics

Data was analyzed using SPSS software (version 10.0) for presentation. Results are given in mean, standard deviation (SD), and range (minimum–maximum). Correlation was done using Spearman rank correlation since self-reported data was ordinal variable. The Spearman correlation coefficient was interpreted as follows: <0.3: none; 0.31–0.5: weak; 0.51–0.7: strong; 0.71–0.9: very strong; and >0.9: excellent [15].

## 3. Results

Table 2 shows mean and standard deviation along with range of all subscales of KOOS and 6-MWT in primary OA knee patients. Overall KOOS-sports and recreation had a minimum mean score followed by QOL subscale. Average, OA knee patients covered 251.77 m in 6 minutes.

Table 3 shows correlation between KOOS and other objective measures. 6-MWT had a weak correlation with KOOS-ADL (rho 0.461) and strong correlation with KOOS-symptom, KOOS-pain, and KOOS-sports (rho 0.578, 0.619, and 0.536, resp.) and very strong correlation with KOOS-QOL (rho 0.733). BMI had a strong correlation with KOOS-pain, KOOS-symptom, KOOS-ADL, and KOOS-sports (rho −0.683, −0.641, −0.523, and −0.640, resp.) and very strong correlation with KOOS-QOL (rho −0.816). Weight had a weak correlation with KOOS-symptom, KOOS-ADL, and KOOS-sports (rho −0.485, −0.333, and −0.413, resp.) and strong correlation with KOOS-pain and KOOS-QOL (rho −0.542 and −0.582, resp.). All the above values were highly significant with *P* < 0.001. There was no correlation between age and any of KOOS subscales except age and KOOS-QOL subscale (rho 0.159, *P* < 0.05).

## 4. Discussion

The aim of the present study was to see the correlation between KOOS five subscales and some of the objective measures, that is, 6-MWT, age, height, weight, and BMI. The results show there is weak (none) correlation of KOOS subscales with height, progressively increase with weight and BMI. KOOS has almost no correlation with age and weak to strong correlation with 6-MWT. Our results are supported by the following studies.

Sabirli et al. [9] mean score of KOOS all subscales were less than present study results but trend is similar between the two studies meaning sports and recreation subscale is the least followed by QOL subscale and symptom subscale was the maximum in both studies.

The present study results are in agreement with Sabirli et al. [9] who reported strong correlation between timed up and go (TUG) and all subscales of KOOS. They reported −0.66, −0.521, −0.531, −0.694, and −0.561 for pain, symptom, ADL, sports, and QOL whereas present results for them to 6-MWT was 0.619, 0.578, 0.461, 0.536, and 0.733, respectively. Liikavainio et al. [11] found strong correlation between WOMAC and 5-MWT (*r*  −0.485, −0.525, and −0.577 for pain, stiffness, function subscales, respectively, *P* < 0.001 for all). Sutbeyaz et al. [16] reported negative correlation between WOMAC pain, function subscales, and 6-MWT (*r*  −0.205 for pain, *P* < 0.001; *r*  −0.646 for function, *P* < 0.05). Similarly, Maly et al. [7] reported mild but significant correlation between 6-MWT, WOMAC pain, and stiffness subscales (*r*  −0.39 for pain and *r*  −0.48 for stiffness). Both Maly et al. [7] and Liikavainio et al. [11] also reported positive correlation between TUG and WOMAC subscales.

Lin et al. [17] found mild correlation between WOMAC and physical performance tests in OA knee patients (*r*  0.33–0.54). Steultjens et al. [1] observed mild but significant correlation between observed and self-reported physical performance (*r*  0.20–0.26; *P* < 0.01). Kennedy et al. [18] found mild to moderate correlation (*r*  0.21–0.50) between self-report and actual physical performance on 1044 knee arthroplasty patients. Stratford et al. [19] reported moderate correlation between self-reported lower extremity function scale (LEFS) and TUG time (*r* 0.42) on patients waiting for knee arthroplasty. Brandes et al. [20] reported positive correlation between gait cycle and SF-36 pain domain (*r* 0.6-0.7, *P* < 0.001) as well as physical function domain (*r* 0.5, *P* < 0.05). Recently, Adegoke et al. [21] reported a positive correlation (*r* = 0.56) between self-reported function and actual physical performance (stair climbing and TUG test) which is similar to our result (*r* = 0.461).

Tanamas et al. [22] in their longitudinal study reported that weight gain is associated with worsening pain, stiffness, and function in knee pain patients. Miller et al. [23] reported that 6 monthly changes in weight is correlated with WOMAC pain (*r* 0.346, *P* < 0.01), stiffness (*r* 0.204), and function (*r* 0.310, *P* < 0.05), respectively. Penserga and Tanque [24] reported a positive correlation between WOMAC (function) and weight (*r* = 0.260).

Correlation between weight and BMI is similar to that of Marks [25] (0.764 in present study versus 0.83 and 0.896 in Marks' study) and their reported *r* = −0.63 for self-determined walking pace with BMI is similar to our KOOS-ADL with BMI (*r* = −0.523). However contradictory to our finding, Marks [25] reported lower values for VAS pain scale with BMI (*r* = 0.352). Aoyagi et al. [26] reported positive association between BMI and knee pain. Marks [27] concluded that BMI was significantly correlated with pain using VAS (*r* 0.270, *P* < 0.01) and disability using AIMS (*r* 0.357, *P* < 0.01).

Elbaz et al. [28] reported BMI significantly correlated with all subscales of WOMAC (*P* < 0.001) and SF-36 but their correlation was <0.30. Previous studies also report similar correlation between BMI and WOMAC (Sanghi et al. [29]; *r* = 0.592, 0.634, 0.749 for pain, stiffness, and ADL subscales in Külcü et al. [30] except Cubukcu et al. [31]. Creamer et al. [32] reported positive correlation (*r* = 0.42) between WOMAC function subscale and BMI. Maly et al. [33] reported mild positive correlation between BMI and sit to stand performance (*r* 0.47, *P* < 0.01).

Marks [27] concluded that age was not significantly correlated with pain using VAS (*r*  −0.122) and disability using AIMS (*r*  −0.175). Penserga and Tanque [24] reported lack of correlation between age and WOMAC disability subscales (*r*  −0.077). Nebel et al. [34] reported lack of correlation between age and objectively measured gait parameters. Lack of correlation between KOOS pain (*r* 0.093), symptom (*r* 0.071), and ADL (*r* 0.036) subscales with age and weak correlation between age and KOOS QOL (*r* 0.159) subscale in OA knee patients have already been published in our previous publication with larger sample size [35]. Age found to be insignificant correlation with WOMAC pain (*R* ^2^ 0.01) and function (*R* ^2^ 0.06) subscales in 257 knee arthroplasty patients [36]. Elbaz et al. [28] also reported similar results in symptomatic OA knee patients using WOMAC (0.028, 0.023, 0.09 for pain, stiffness, and function subscales, resp.). Cubukcu et al. [31] reported similar result using WOMAC (correlations between age and WOMAC pain, stiffness, and function were 0.081, −0.49, 0.114, and resp.; all were statistically insignificant). Recently, Maly et al. [33] in their study found insignificant correlation between age and pain intensity (*r*  −0.03) and physical performance (*r* 0.09).

## 5. Conclusion

KOOS is strongly positively correlated with 6-MWT and negatively correlated with BMI. Its correlation strength has decreased with weight. The present study results are in confirmation with previous studies and affirms the usage of KOOS in Indian OA knee patients.

## Figures and Tables

**Table 1:** Anthropometric characteristics of patients (*n* = 251).

Character	Mean	Standard deviation (SD)	Range (minimum–maximum)
Age (in yrs)	56.52	8.90	41–80
Height (in cm)	161.87	7.20	140–180
Weight (in kg)	84.07	11.24	56–114
BMI (kg·m^−2^)	32.11	4.01	23.03–43.37

**Table 2:** 6-MWT and KOOS scores among clinical OA knee patients (*n* = 251).

Parameter	Mean	Standard deviation (SD)	Minimum–maximum
KOOS-pain (in %)	62.90	18.05	11–94
KOOS-symptom (in %)	69.15	16.82	32–96
KOOS-ADL (in %)	67.27	18.39	9–97
KOOS-sports/recreation (in %)	36.31	22.91	0–95
KOOS-QOL (in %)	46.58	20.75	13–94
6-MWT (in meters)	251.77	35.04	170.5–337

**Table 3:** Correlation between KOOS and other objective measures (*n* = 251).

	Age	Height	Weight	BMI	6-MWT
KOOS-pain	0.093	0.136*	−0.542***	−0.683***	0.619***
KOOS-symptom	0.071	0.167**	−0.485***	−0.641***	0.578***
KOOS-ADL	0.036	0.223***	−0.333***	−0.523***	0.461***
KOOS-sports	0.028	0.272***	−0.413***	−0.640***	0.536***
KOOS-QOL	0.159*	0.268***	−0.582***	−0.816***	0.733***

^∗,∗∗,∗∗∗^Means *P* < 0.05, *P* < 0.01, and *P* < 0.001, respectively.
